# Normal perinatal and paediatric postmortem magnetic resonance imaging appearances

**DOI:** 10.1007/s00247-014-3166-y

**Published:** 2015-04-01

**Authors:** Owen J. Arthurs, Joy L. Barber, Andrew M. Taylor, Neil J. Sebire

**Affiliations:** 1 Department of Radiology, Great Ormond Street Hospital for Children NHS Foundation Trust, Great Ormond Street, London, WC1N 3JH UK; 2 UCL Institute of Child Health, London, UK; 3 Cardiorespiratory Division, Great Ormond Street Hospital for Children NHS Foundation Trust, London, UK; 4 Centre for Cardiovascular Imaging, UCL Institute of Cardiovascular Science, London, UK; 5 Department of Histopathology, Great Ormond Street Hospital for Children NHS Foundation Trust, London, UK

**Keywords:** Magnetic resonance imaging, Autopsy, Pathology, Fetus, Children

## Abstract

As postmortem imaging becomes more widely used following perinatal and paediatric deaths, the correct interpretation of images becomes imperative, particularly given the increased use of postmortem magnetic resonance imaging. Many pathological processes may have similar appearances in life and following death. A thorough knowledge of normal postmortem changes is therefore required within postmortem magnetic resonance imaging to ensure that these are not mistakenly interpreted as significant pathology. Similarly, some changes that are interpreted as pathological if they occur during life may be artefacts on postmortem magnetic resonance imaging that are of limited significance. This review serves to illustrate briefly those postmortem magnetic resonance imaging changes as part of the normal changes after death in fetuses and children, and highlight imaging findings that may confuse or mislead an observer to identifying pathology where none is present.

## Introduction

As postmortem imaging becomes more widely used following perinatal and paediatric deaths, the correct interpretation of images becomes imperative, particularly given the increased use of postmortem magnetic resonance imaging. Whilst the diagnostic accuracy of postmortem magnetic resonance imaging, in general, has been shown to be high following specialist interpretation [1], post mortem magnetic resonance imaging is prone to errors made by misinterpretation of normal postmortem changes as pathology, and vice versa [2].

Practitioners of paediatric and perinatal postmortem magnetic resonance imaging will need expertise in several areas, including knowledge of foetal and congenital abnormalities, the range of perinatal pathologies encountered, awareness of general paediatric radiologic principles and an understanding of specialist MR techniques. As with all new imaging domains, there are several potential pitfalls and errors that can be made during postmortem magnetic resonance imaging interpretation, not only in differentiating the normal postmortem changes from pathology, but also recognizing several postmortem changes, which may be incorrectly interpreted as pathology or requiring further investigation. Here, we illustrate postmortem magnetic resonance imaging changes that occur normally after death in fetuses and children, and highlight imaging findings that may confuse or mislead an observer to identifying pathology where none is present, from our experience of more than 500 postmortem magnetic resonance imaging examinations at a specialist children’s hospital.

## Normal changes after death

There are changes that occur in a body following death, either in utero or during the interval from death to autopsy, which result in features considered to be normal postmortem changes. Most of these are well recognised by paediatric pathologists on direct visual examination, but the imaging correlates of these need further characterisation.

**Fig. 1:**
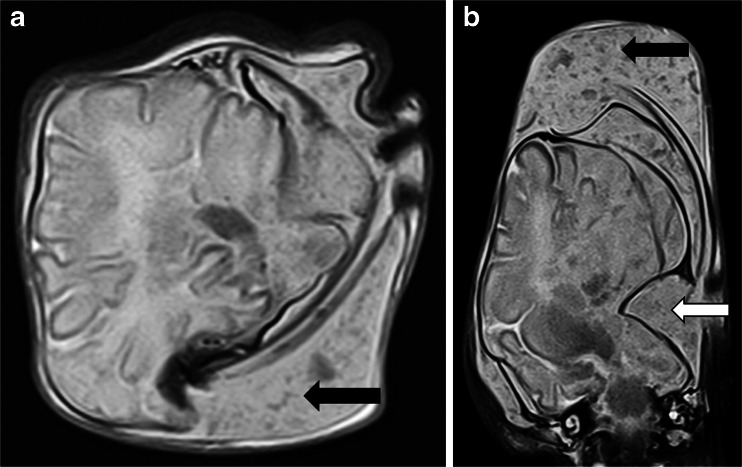
Severe head moulding. T2-weighted postmortem magnetic resonance imaging brain imaging of a 39-week gestation fetus with severe disruption of the skull following delivery, making both axial (**a**) and coronal (**b**) imaging difficult to interpret. There is soft tissue oedema (*black arrow*) as well as disruption of the skull, primarily along the sutures (*white arrow*). The intracranial appearances were normal at autopsy

**Fig. 2:**
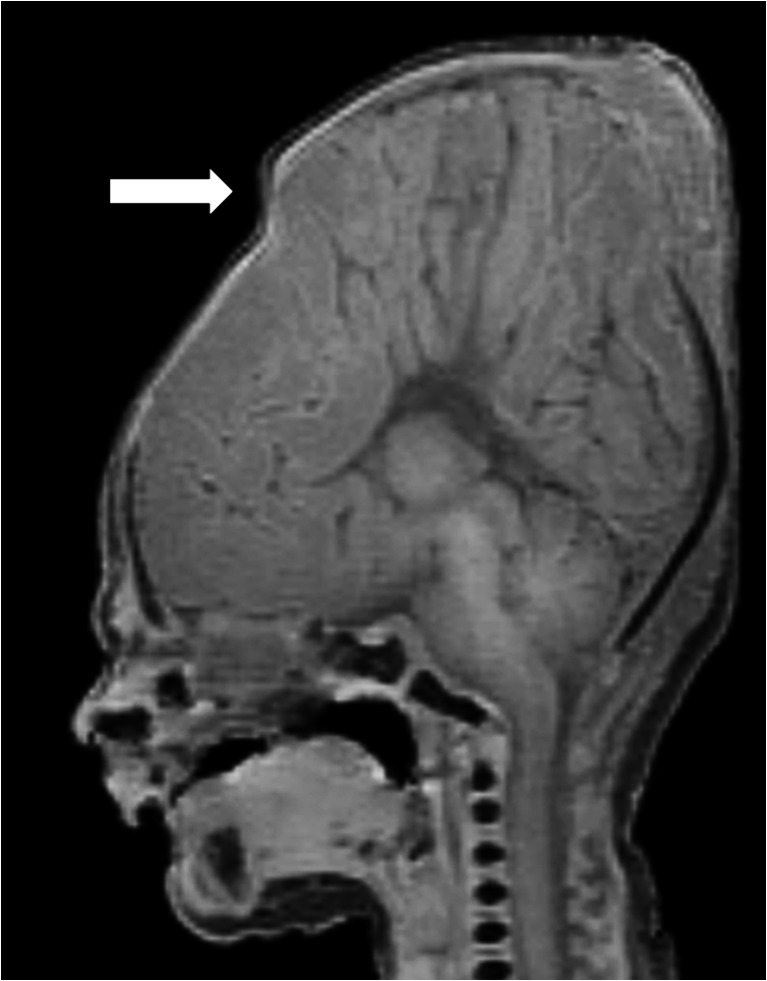
Head moulding. T1-weighted postmortem magnetic resonance imaging sagittal brain image of a 34-week gestation fetus with significant skull and head moulding, with separation of the sutures (*white arrow*) likely secondary to delivery. There was agenesis of the corpus callosum, confirmed at autopsy

**Fig. 3:**
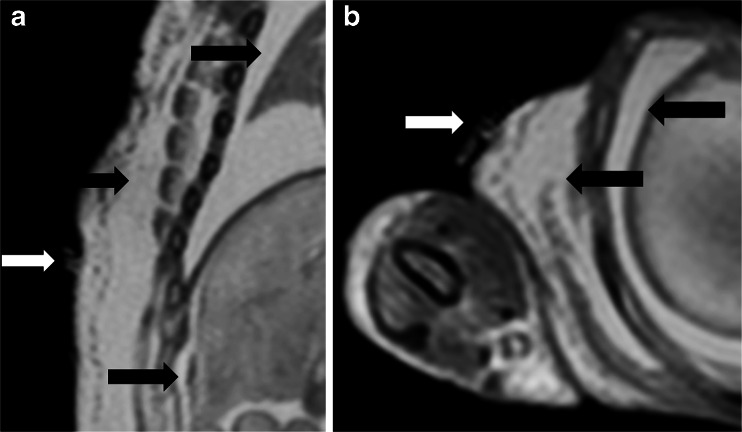
Maceration. Coronal (**a**) and axial (**b**) T2-weighted postmortem magnetic resonance imaging body imaging of a 26-week gestation fetus with skin slippage due to maceration. Skin fragments can be seen on the body surface (*white arrows*) with significant underlying subcutaneous oedema around the upper limb and thorax, a large pleural effusion and ascites (*black arrows*)

The main process that occurs following death is termed autolysis, which represents cellular breakdown of body tissues, probably enzyme driven, leading to changes in tissue structure and permeability and fluid redistribution throughout body compartments [3]. The imaging correlations of this are frequently encountered, but the rate at which such changes occur is poorly understood and probably depends upon several factors including antemortem circumstances, age, mode of death, geographical location of death, body storage conditions including temperature and a host of other factors. In addition to autolysis, simultaneous putrification also occurs secondary to bacterial colonisation, which may result in further tissue breakdown and gas formation. In routine paediatric practice, additional factors such as the effects of scavengers and insect activity will be rarely encountered and will not be considered here. Approximate estimates of time of death are often based on these overall changes [3]. Unfortunately, foetal postmortem changes may be further complicated by in utero death with a period of retention prior to delivery, in which there is softening of the fetus within fluid (in this case, amniotic fluid), a process termed maceration. Maceration-related changes are well-recognised by the pathologist on external examination as skin slippage, skin and umbilical cord discoloration, followed by more extensive discoloration and liquification of cranial contents with separation of the sutures. Extensive intrauterine retention after death leads to mummification. These changes are also encountered in imaging studies and can give rise to a range of typical appearances including loss of skull integrity with extreme changes in head shape (Fig. 1), which should be differentiated from head moulding that occurs at delivery (Fig. 2), and skin slippage (Fig. 3), which mimics other changes.

The degree of maceration has been used to estimate the time the fetus has been retained [4, 5], and it may be possible to derive similar features based on postmortem imaging criteria. Since the exact time of in utero death is usually difficult to establish with certainty in most cases of intrauterine death because of a reliance on maternal recognition (e.g., reduced foetal movements), the precise imaging correlates of the extent of maceration are difficult to quantify. This process is further complicated by the fact that the technique of fetocide significantly alters the rate of postmortem tissue degradation, particularly when potassium chloride is used [6]. This means that cases undergoing termination of pregnancy by fetocide with precisely known timing cannot be extrapolated to spontaneous deaths [6, 7]. Histological appearances of foetal tissues can also be used to assess the extent of maceration changes, such as the degree and location of loss of nuclear basophilia and cellular breakdown in different tissues [5]. However, these measures only give an estimation of length of retention following intrauterine death and there is limited data regarding how such features are affected by other variables and the superimposed postmortem interval.

**Fig. 4:**
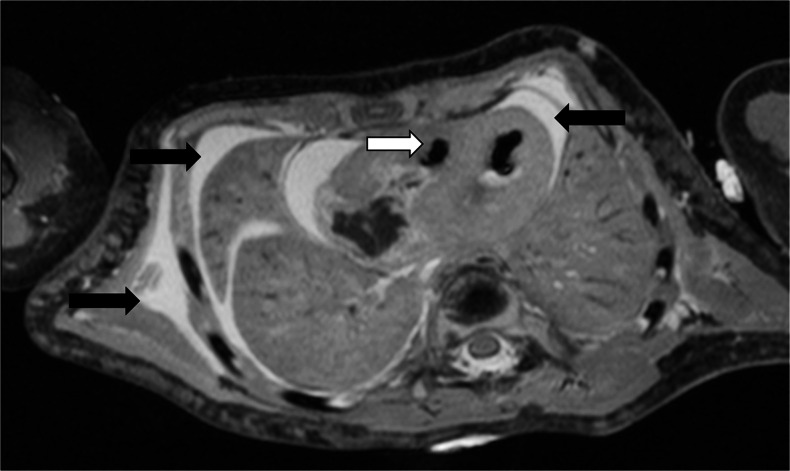
Fluid redistribution. Axial high-resolution T2-weighted postmortem magnetic resonance imaging in a term stillbirth. This demonstrates postmortem subcutaneous oedema, pleural and pericardial effusion (*black arrows*), and intracardiac gas (*white arrow*)

**Fig. 5:**
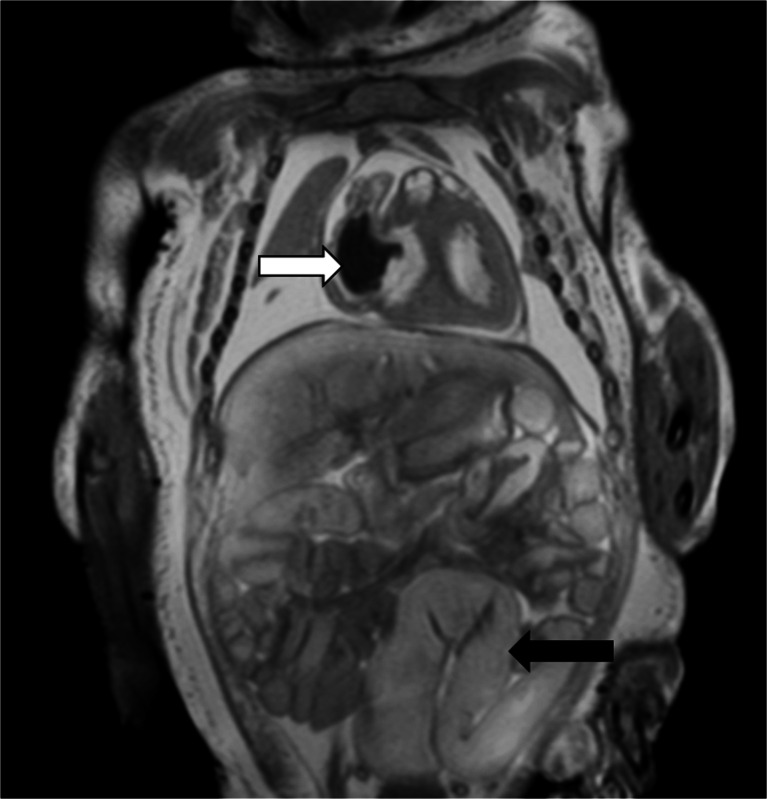
Fluid redistribution. Coronal T2-weighted postmortem magnetic resonance imaging in a 31-week stillbirth. This demonstrates postmortem subcutaneous oedema, moderate pleural effusion, intracardiac gas (*white arrow*) and abdominal bowel dilatation (*black arrow*), which can all be misinterpreted as pathological

**Fig. 6:**
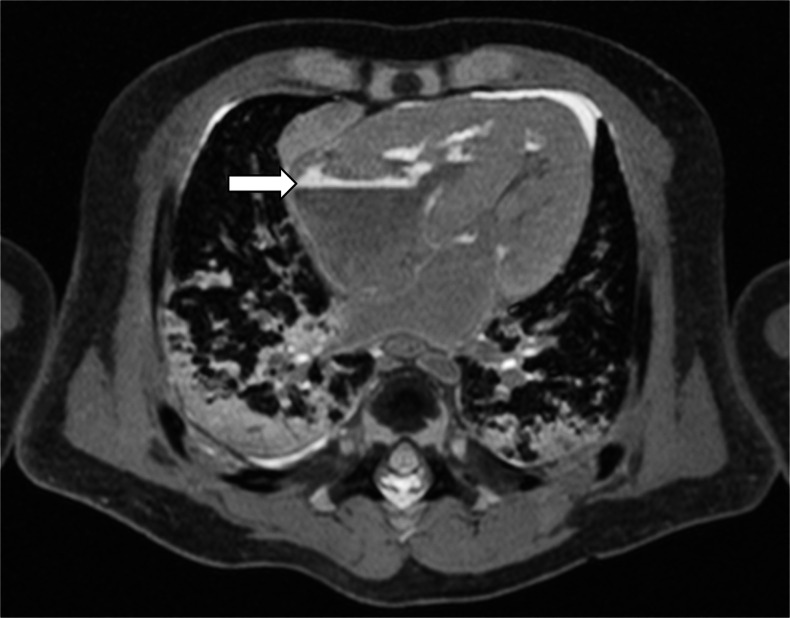
Dependent lung changes. Axial high-resolution T2-weighted postmortem magnetic resonance imaging in a term stillbirth. There are dependent changes in both lungs, which are a normal finding. There is also normal postmortem sedimentation or layering of blood in the heart, shown as a fluid-fluid level (*white arrow*). Note that by imaging earlier in the postmortem interval in this case, fluid redistribution and gas in the heart may not yet have accumulated (compared to Figs. 4 and 5)

Autolysis, and maceration where appropriate, is likely to account for several of the changes of fluid accumulation that are observed with cellular breakdown. In the postmortem fetus and child, fluid normally accumulates in the subcutaneous tissues, pleural space, pericardial sac and peritoneal cavity (Figs. 4 and 5), none of which represents a pathological process in most cases. The relative distribution of fluid between body compartments is usually evenly distributed and typically gravity dependent; fluid accumulation in one compartment out of proportion to the others may be considered pathological. For example, together with difficulties in interpreting postmortem changes in the lungs (Fig. 6), pleural effusions could be interpreted as parapneumonic effusions (and may therefore suggest a cause of death), whereas effusions in keeping with the degree of fluid redistribution elsewhere in the subcutaneous tissues and ascites is likely to be non-pathological. Foetal hydrops can therefore sometimes be difficult to discriminate from normal postmortem changes, and it may be only the antemortem history or antenatal US examinations that may suggest the difference.

## Changes in positioning after death

Some of the most frequent changes encountered when imaging after death relate to body position, including slumping or organ shifting (soft tissue laxity) and rigor mortis in non-foetal cases. Fixed flexion deformities due to stiffening of the limbs and joints in rigor mortis make positioning the body appropriately for imaging challenging, particularly for post mortem paediatric radiography. Some methods of overcoming this involve imaging in different positions or taking multiple radiographs at different angles to highlight different aspects of the long bones [8]. Apparent fixed flexion deformities on both postmortem radiography and postmortem magnetic resonance imaging can be misinterpreted as arthrogryphosis multiplex congenita and vice versa, whereas oligohydramnios and rigor mortis in otherwise normal fetuses can give similar appearances. It is therefore essential that imaging findings are interpreted in conjunction with the clinical history and macroscopic external appearances.

**Fig. 7:**
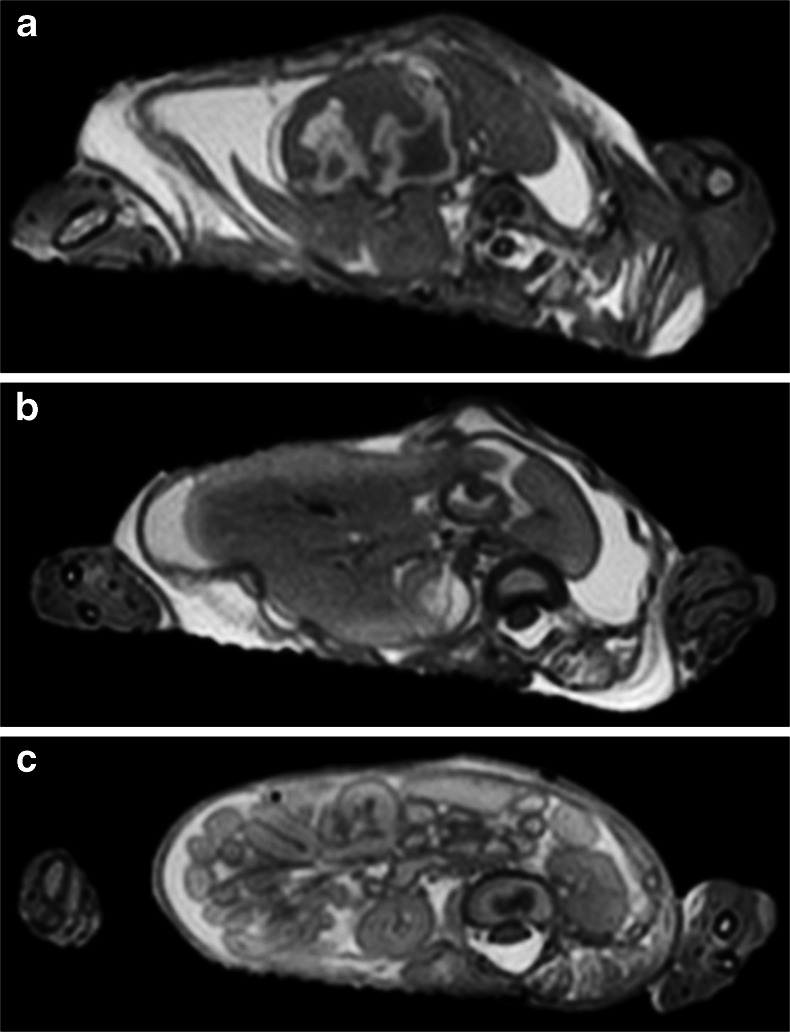
Body organ shifting. Axial T2-weighted postmortem magnetic resonance imaging of a 27-week gestation unexplained intrauterine death, demonstrating organ shifting of the body to one side, affecting the thoracic (**a**) and abdominal (**b** and **c**) compartments. This leads to overinterpretation and misdiagnosis: Both malrotation and asplenia were initially reported in this case prior to 3-D reformatting, as the spleen was difficult to visualise separate from the liver (**b**) and small bowel loops were predominantly right-sided (c). No anatomical abnormality was identified at full autopsy in this example. Subcutaneous oedema, but no skin slippage, is apparent

With increased soft tissue laxity and muscle relaxation in the period immediately after death, body organs often slump or shift into non-physiological positions due to the effect of gravity. Imaging bodies in slumped positions can lead to significant misinterpretation of normal anatomy. This can lead to some organs being difficult to identify accurately as they are not in their normal anatomical position (e.g., the spleen, or proximal duodenum, suggesting malrotation in Fig. 7), or simply make accurate assessment of the mediastinal structures difficult secondary to deviation (Fig. 7).

## Organ-specific postmortem changes

### Intracranial contents

**Fig. 8:**
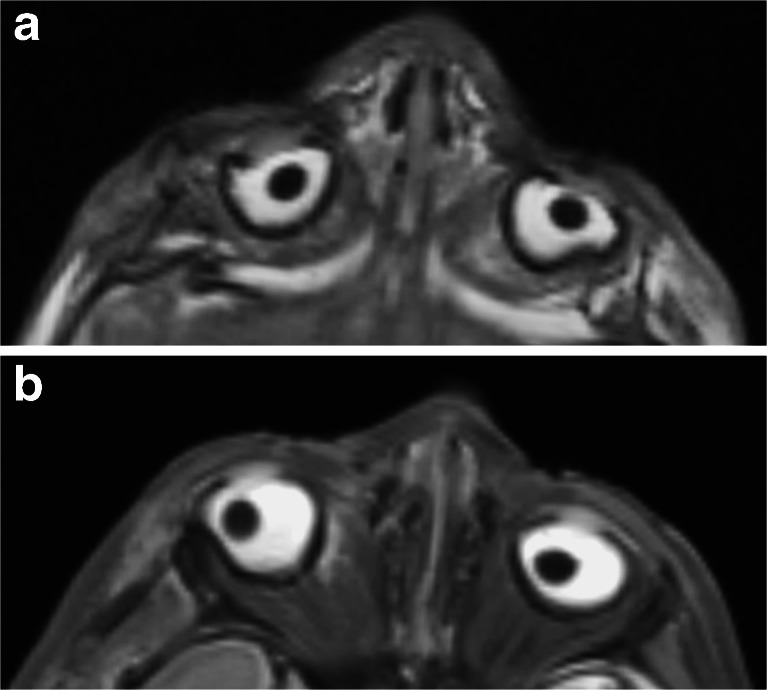
Ocular changes. Two examples of axial T2-weighted postmortem magnetic resonance imaging images from late gestation fetuses show collapse of the globes (**a**), with dislocation of the lens (**b**). These are normal postmortem changes that occur in the majority of cases, due to lack of fluid in the globe, and may be mistaken for pathology. However, lens dislocation does not always occur (see Fig. 10)

**Fig. 9:**
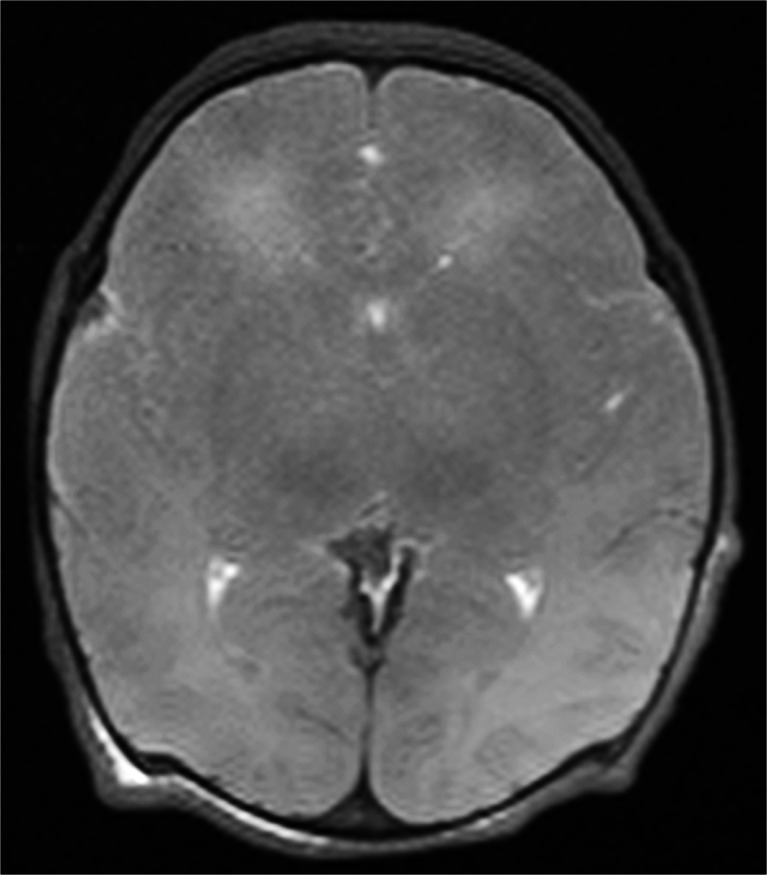
Brain ischaemia. Axial T2-weighted postmortem magnetic resonance imaging of the brain in a term baby found abandoned. There is gross cerebral oedema with loss of sulcal definition, and almost complete loss of the grey-white matter interface. It is not currently possible to differentiate antemortem from postmortem hypoxic-ischaemia brain injury on conventional postmortem magnetic resonance imaging sequences

Extensive skull deformation (Fig. 1) and sunken globes (Fig. 8) are normal postmortem changes following perinatal death, but they can make interpretation of brain imaging findings difficult. Furthermore, even to the experienced neuroradiologist, one of the most difficult diagnoses to make on postmortem magnetic resonance imaging is to differentiate antemortem from postmortem hypoxic-ischaemic cerebral injury [1]. Brain ischaemia is a common feature of death, whether a primary hypoxic event was the cause or whether secondary hypoxia has occurred following circulatory collapse. A combination of diffuse brain swelling/oedema (with effacement of sulci), loss of grey-white matter differentiation and abnormal increase in signal intensity of deep grey matter structures (basal ganglia and thalami) and cerebellum are markers of global brain ischaemia in life, and can be applied following death (Fig. 9), but their clinical significance postmortem remains unclear. Apparent tonsillar descent is also a frequent normal post mortem finding, secondary to cerebral oedema.

A recent study reported that both T1- and T2-weighted signal changes occur after death, leading to convergence of the grey-white matter signal, but that these apparent ischaemic-type changes could not be differentiated from true antemortem hypoxic changes [9]. A further study in adults has suggested that apparent diffusion coefficient (ADC) values on diffusion-weighted MRI may be subtly different depending on the cause of death (higher white matter ADC in heart failure, lower in hypoxia due to strangulation or traumatic death [10]), although this has yet to be replicated in the perinatal scenario.

**Fig. 10:**
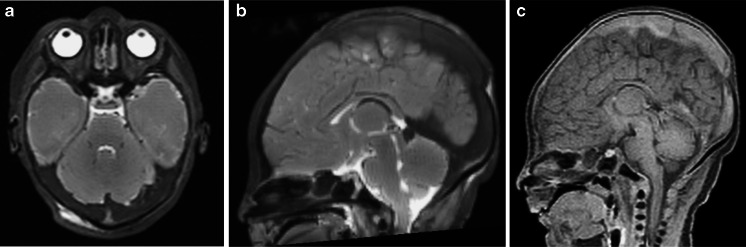
Venous sinus thrombosis. Axial (**a**) and sagittal T2-weighted (**b**) imaging, and sagittal T1-weighted (**c**) postmortem magnetic resonance imaging imaging of the brain in a neonatal death. These images show thrombosis of almost the entire venous sinuses, which is typically seen postmortem (rather than sedimentation as in Fig. 13). It is not currently possible to distinguish antemortem from postmortem venous sinus thrombosis. Note the absence of lens dislocation, which does not always occur (see Fig. 8)

**Fig. 11:**
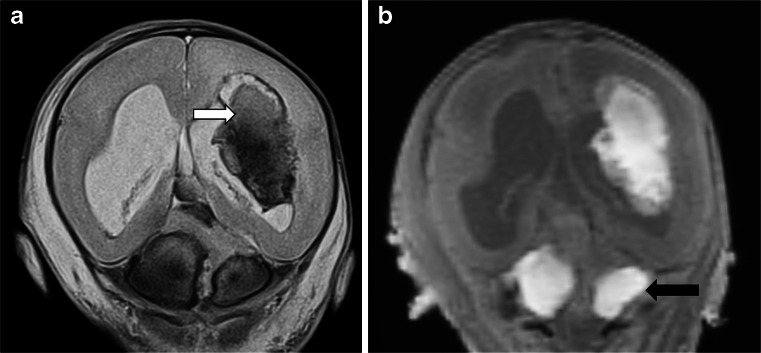
Intracranial haemorrhage. Coronal T2-weighted (**a**) postmortem magnetic resonance imaging imaging of the brain at 25 weeks, showing haemorrhage in the left lateral ventricle with parenchymal haemorrhage (*white arrow*), and blood in the basal cistern (*black arrow*), which is high signal on T1-weighted images (**b**). This degree of haemorrhage is clearly pathological, but small intraventricular haemorrhages in fetuses may occur spontaneously

Venous stasis is a normal finding following death, but can equally also be misintepreted as antemortem venous thrombus (Fig. 10). More accurate characterisation of the rate at which these changes occur, together with knowledge of the antemortem state, body preservation techniques and time interval from death to imaging, may help to discriminate these postmortem changes from antemortem or perimortem pathology. T1-weighted imaging may be used to highlight haemorrhagic parenchymal changes (Fig. 11), and more advanced MR techniques such as gradient-echo susceptibility weighted imaging [11] need to be evaluated in this context. Similarly, small intraventricular haemorrhages in fetuses may be interpreted as normal spontaneous events [1].

### Thorax

**Fig. 12:**
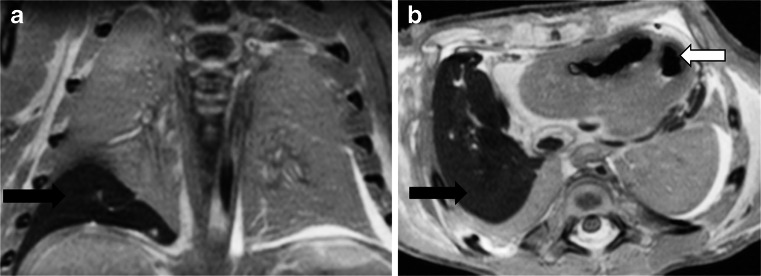
Lung changes after fetocide. Coronal (**a**) and axial (**b**) high-resolution T2-weighted postmortem magnetic resonance imaging following termination at 35 weeks’ gestation. Very low signal was noted in the right lower lobe (*black arrows*), with minimal pericardial effusion but intracardiac gas (*white arrow*). This was interpreted as signal change related to fetocide, which was performed by intracardiac injection of potassium chloride (which may account for the air in the heart), and the needle may have traversed the right lower lobe. However, there was no macro- or microscopic difference between right and left lower lobe at autopsy

Postmortem magnetic resonance imaging images of the lungs are difficult to interpret, even for experienced paediatric radiologists [1]. Lungs with unequivocal pathology such as pneumonia with sepsis can appear normal on postmortem magnetic resonance imaging, particularly in older children. Conversely, it is also possible to overinterpret normal non-uniform fluid accumulation in both parenchyma and interstitium as pathology, when it most likely represents normal postmortem change (Fig. 4). Furthermore, several different types of pathology can give similar parenchymal appearances, such as aspiration, haemorrhage, infection or other pathology, or artefact (Fig. 12).

One approach to overcome this would be to ventilate the lungs after death. Using a portable ventilator to generate positive airway pressure can cause sufficient lung expansion and reduction in lung density on postmortem computer tomography to help discriminate true pathology from postmortem change. This ventilated postmortem CT has been shown to be useful in adults [12] but not yet assessed in children.

**Fig. 13:**
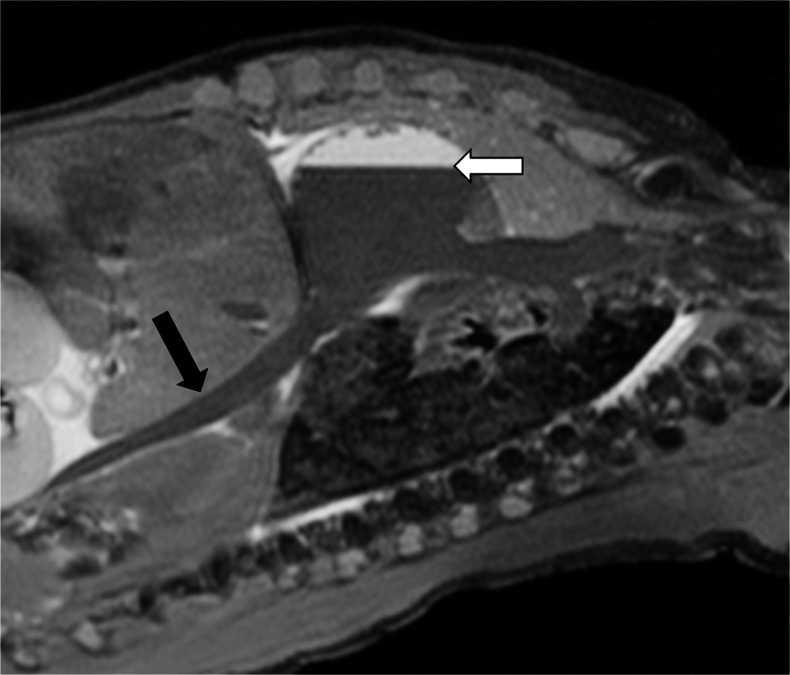
Blood layering. Sagittal T2-weighted postmortem magnetic resonance imaging in a term stillbirth. There is gravity = dependent sedimentation or layering of blood in the heart, shown as a fluid-fluid level (*white arrow*), which is frequently seen. Note gradual tapering and collapse of the inferior vena cava due to abdominal organ compression (*black arrow*), which is not pathological

Stasis of blood for prolonged periods after death leads to sedimentation of the cellular from the plasma contents, giving rise to fluid-fluid levels in the major vessels and heart (Figs. 6 and 13). As this is a normal finding, it is important not to diagnose thrombus or pulmonary embolus as a cause of death [13, 14]. It remains unclear as to how quickly these changes occur and whether true thrombus can be accurately identified on perinatal postmortem imaging.

**Fig. 14:**
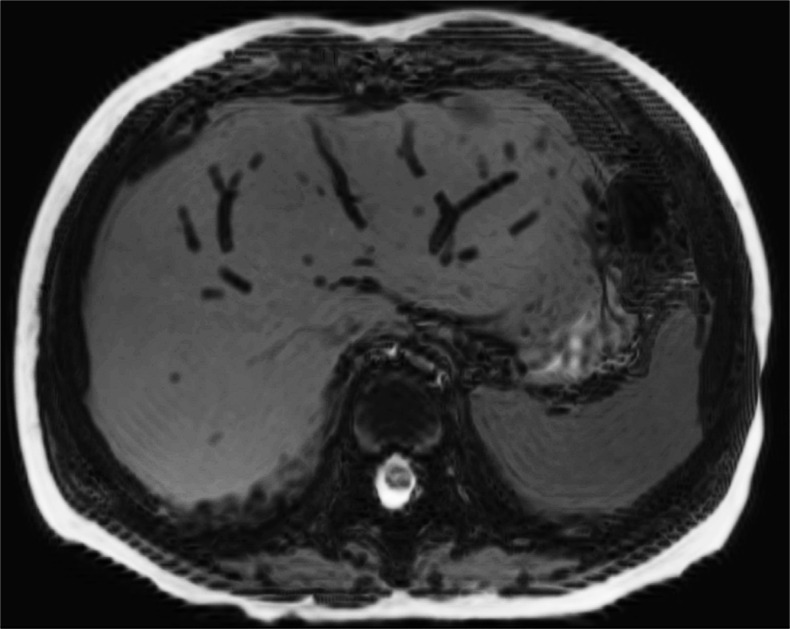
Hepatobiliary gas. Axial minimum-intensity projection T2-weighted postmortem magnetic resonance imaging image following neonatal death shows hepatobiliary air, likely in the hepatic veins in this case

Equally, there has been some debate about the origin of the sometimes significant quantities of gas seen throughout the body on postmortem imaging, with theories regarding gas originating from putrefaction, trauma or resuscitation efforts [15, 16]. Although gas in the hepatobiliary system and intravascular gas are often seen at postmortem magnetic resonance imaging (Fig. 14), these features may not be attributable to any cause (e.g., gas embolus) with any diagnostic certainty.

### Abdomen

**Fig. 15:**
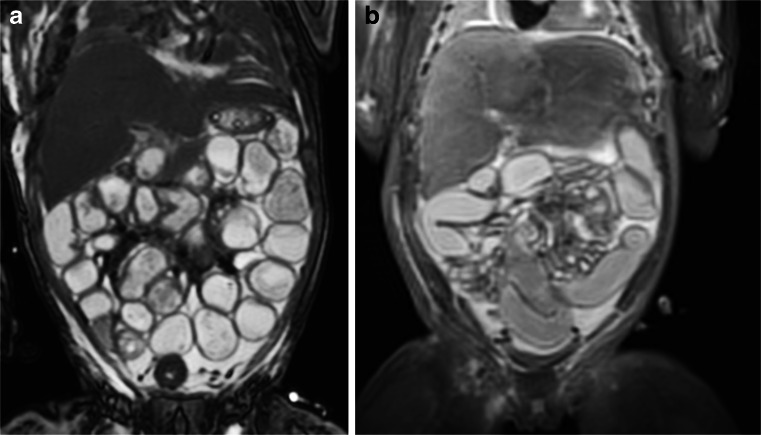
Bowel dilatation. Two examples of coronal T2-weighted postmortem magnetic resonance imaging images from neonatal deaths, which show different degrees of bowel dilatation (left **a** more severe than right **b**), which were interpreted as being bowel obstructions. In both cases, these represent normal postmortem dilatation of the bowel, and the bowel was normal at autopsy

Whilst postmortem magnetic resonance imaging is particularly good at identifying certain abdominal pathologies, such as renal cystic disease and obstructive uropathy, there are several difficult areas in abdominal postmortem magnetic resonance imaging imaging. Whilst Meckel diverticulum and abdominal malrotation can be missed on imaging in small fetuses, the main issue in abdominal imaging is discriminating pathological small and large bowel dilatation from normal postmortem change. To the paediatric radiologist, several cases of bowel dilatation may be interpreted as malrotation or lower GI obstruction, stenosis or atresia, as they occur in life, whereas at autopsy dilated bowel loops simply represent normal postmortem changes (Fig. 15).

**Fig. 16:**
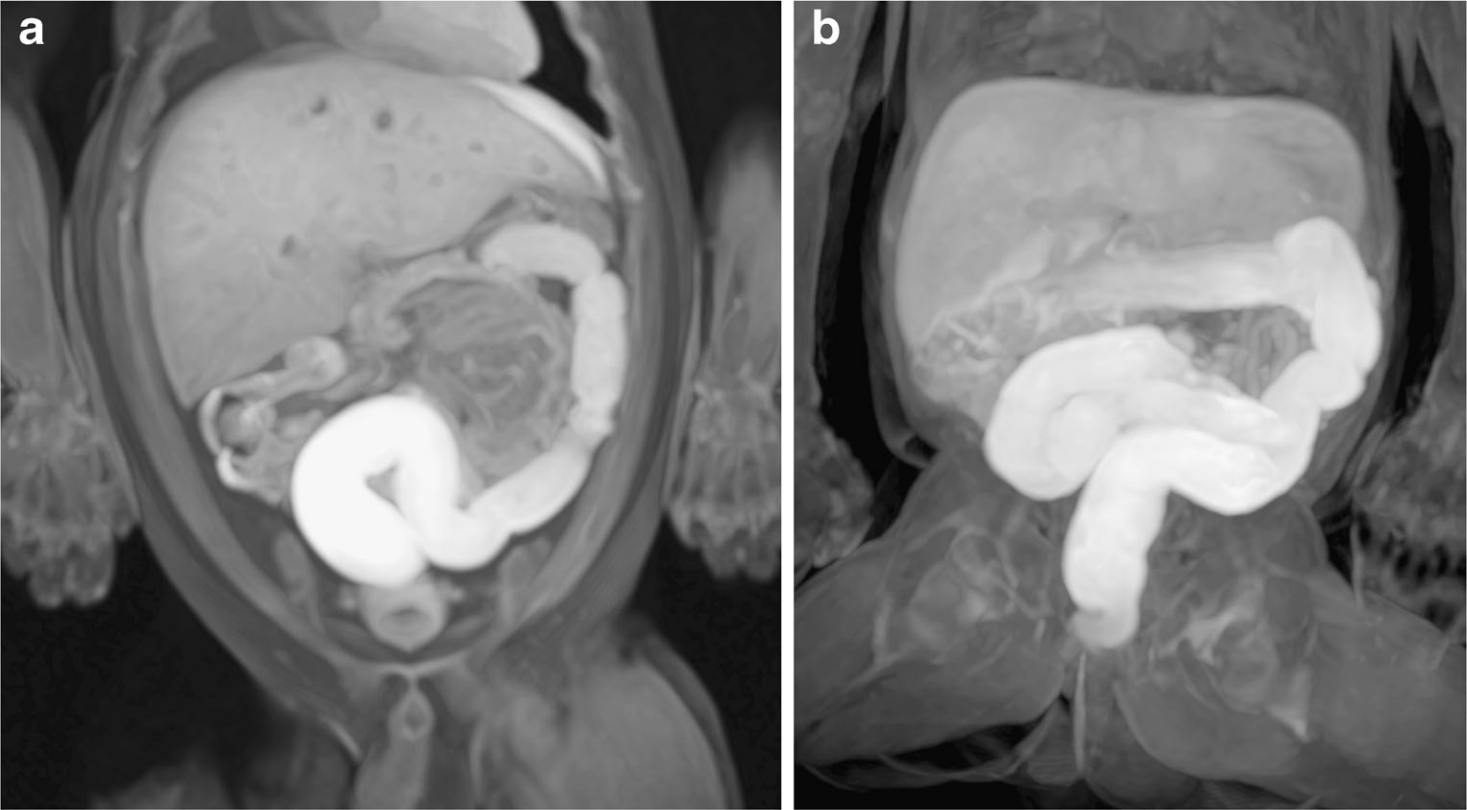
Normal meconium. Two examples of coronal T1-weighted VIBE MPR (volume interpolated breathhold examination multiplanar reformat) postmortem magnetic resonance imaging images from neonatal deaths, which show the normal gradually increasing high signal within the distal large bowel, consistent with normal meconium, similar to those seen on foetal or prenatal MRI. This can occur either predominantly from the sigmoid colon onwards (**a**) or from more proximal colon (**b**) The liver is of high signal on T1-W images (as opposed to low signal on T2-W images, e.g., Fig. 15)

Gaseous distension of the bowel appears to occur rapidly following perinatal death, and unless there is a clear transition point, should not be interpreted as pathological. At autopsy, postmortem dilatation of the bowel can be discriminated from obstructive or ischaemic causes by examination of the entire gastrointestinal tract by direct observation, sampling and examination of any macroscopically abnormal areas. Marked bowel dilatation of otherwise normal gas-filled bowel loops, especially in infants and children, is a recognized non-specific feature at autopsy and does not, in isolation, indicate obstruction as an underlying cause of death. However, sudden unexpected deaths due to bowel obstruction may occur in this age group [17]. T1-weighted imaging can highlight the normal distribution of meconium (Fig. 16), just as it does in antenatal/foetal MR [18], and may be a useful feature in some circumstances. Autolysis of the gastric wall rarely leads to spontaneous perforation, which may be misinterpreted as antemortem gastric perforation [19].

### Age-related changes

Apart from in utero maceration, there may be a host of other factors that influence the rate at which these described normal postmortem changes occur, as well as how appearances of distinct pathological changes are modified at different ages. At present, there is insufficient data to reliably determine the relative rates of such postmortem changes by age and underlying pathology, but our appreciation of imaging-related changes will be improved by rigorous ongoing studies into the interaction between age, gestation, mode of death and postmortem interval in different settings.

## Conclusion

The recognition of normal postmortem changes as well as imaging-related artefacts are crucial for the accurate interpretation of perinatal and paediatric postmortem magnetic resonance imaging. Better understanding of the normal changes that occur after death may significantly improve the yield of postmortem magnetic resonance imaging in children in the future. Only through clinical-radiological-pathological correlation will these changes be thoroughly understood, and they are likely to require a collaborative approach among foetal medicine specialists, perinatal pathologists and paediatric radiologists on local, national and international levels.
